# Bibliometric Mapping of Research Trends and Hotspots of Microglia in Spinal Cord Injury (2000–2024)

**DOI:** 10.1002/brb3.70881

**Published:** 2025-09-23

**Authors:** Ziming Cai, Gongpeng Xiong, Jintao Wu, Hanjun Zhang, Jian Huang, Qinghe Yu, Wenping Lin

**Affiliations:** ^1^ Guangzhou University of Chinese Medicine Shenzhen China; ^2^ Department of Hepato‐Biliary‐Pancreatic and Vascular Surgery The First Affiliated Hospital of Xiamen University Xiamen China; ^3^ Department of Orthopedics Haikou Hospital of Traditional Chinese Medicine Haikou China; ^4^ Department of Spine Surgery Shenzhen Pingle Orthopedic Hospital Affiliated Hospital of Guangzhou University of Chinese Medicine Shenzhen China

**Keywords:** bibliometrics, CiteSpace, microglia, spinal cord injury, VOSviewer

## Abstract

**Introduction:**

Spinal cord injury (SCI), acknowledged as the most severe complication arising from spinal trauma, pertains to the dysfunction of the spinal cord due to traumatic events or other pathological conditions. Extensive research has elucidated a substantial correlation between SCI and inflammatory processes, highlighting the critical involvement of microglia in orchestrating neuroinflammatory responses. Moreover, a growing body of evidence has demonstrated a strong connection between microglial activation and both the pathogenesis and progression of SCI.

**Objective:**

We chose bibliometric analysis to comprehensively summarize the research progress of microglia in SCI, aiming to provide researchers with current trends and future research directions.

**Methods:**

All articles and reviews addressing microglia in SCI were systematically retrieved from the Web of Science Core Collection database, spanning publications from 2000 to 2024. Subsequent bibliometric analysis was conducted utilizing four analytical tools: VOSviewer (version 1.6.20), R software (package bibliometrix), the Biblioshiny web interface, and CiteSpace (version 6.2.R4), ensuring comprehensive examination of publication patterns and research trends.

**Results:**

A total of 2428 publications were ultimately included in this bibliometric analysis. The annual publication count demonstrated a consistent upward trajectory. China is the country with the most published articles, and Ohio State University ranks first in institutional publications. *Experimental Neurology* is the journal with the most published articles, while *Journal of Neuroscience* is the journal with the most cited articles. Popovich Pg is the author with the highest productivity and co‐citation. Cluster analysis yielded a total of 15 different co‐citation clusters. Time analysis shows explosive citation outbreaks in 2006, 2009, and 2011. Keyword analysis revealed inflammation, expression, activation, and central nervous system as the most frequently occurring terms. Recent keyword trends feature emerging terms like exosomes, extracellular vesicles, and nanoparticles. Keyword bursts revealed promotes, extracellular vesicle, recovery, neuroinflammation, therapy, polarization, and pathway are the hotspots of research at the present stage and are likely to continue. These findings provide critical insights for developing microglia‐targeted therapeutic strategies and prioritizing research directions in neuroinflammatory modulation to improve functional recovery after SCI.

**Conclusion:**

Emerging research frontiers prominently feature exosomes, gut microbiota, and nanoparticles. The interplay between microglia‐mediated neuroinflammation and SCI has emerged as a critical focal point in current scientific investigations and is anticipated to remain central to forthcoming scientific inquiries.

## Graphical abstract

This bibliometric analysis offers the first thorough overview of hot spots and research trends in microglia in spinal cord injury over the previous 25 years, providing researchers with new ideas in the field. Note that “exosomes,” “gut microbiota,” and “nanoparticles” may be the focus of future studies.

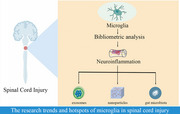

## 1 Introduction

Spinal cord injury (SCI) is a catastrophic neurological disorder that disrupts motor, sensory, and autonomic functions, often leading to lifelong disability (Eli et al. 2021). Epidemiological data show that the global prevalence of SCI has steadily increased over the past 30 years, and the incidence rate of SCI in various countries ranges from 236 cases to 1298 cases per million people (Jiang et al. 2025). Traumatic causes (such as car accidents and falls) and nontraumatic causes (such as degenerative diseases and tumors) have brought increasing burden to the medical system (Bloom et al. 2019). According to data from the National Spinal Cord Injury Statistical Center, the healthcare costs during the first year following injury for an individual with high tetraplegia exceed $1.06 million (Huang et al. 2024). The pathophysiology of SCI is biphasic: The initial mechanical trauma triggers necrotic cell death and axonal shearing (primary injury), followed by a prolonged secondary phase involving ischemia, excitotoxicity, oxidative stress, and neuroinflammation (Venkatesh et al. 2019; Kjell and Olson 2016). It is during this secondary phase that microglia—the CNS‐resident macrophages—play a central yet paradoxical role, driving both degenerative and reparative processes (Sun et al. 2023).

Microglial activation is one of the earliest cellular responses to SCI (Pang et al. 2022). Within minutes of injury, microglia undergo morphological and functional shifts, transitioning from a surveillant ramified state to an amoeboid phenotype capable of phagocytosing debris and releasing cytokines (W. Wu et al. 2024). Historically, activated microglia were viewed as uniformly detrimental, perpetuating neurotoxicity via pro‐inflammatory mediators such as TNF‐α, IL‐1β, and reactive oxygen species (Freyermuth‐Trujillo et al. 2022). However, new evidence highlights their functional plasticity: Subpopulations of microglia adopt anti‐inflammatory and pro regenerative phenotypes (such as M2 like polarization), supporting tissue repair through neurotrophic factor secretion (such as BDNF and IGF‐1), synaptic remodeling, and inflammation resolution (Xu et al. 2021; Timofeeva et al. 2025). This duality underscores the complexity of microglial biology in SCI and complicates therapeutic strategies aimed at modulating their activity (X. Hu et al. 2015).

The past two decades have seen transformative advances in understanding microglial heterogeneity and spatiotemporal dynamics post‐SCI. Techniques such as single‐cell RNA sequencing, fate‐mapping models, and intravital imaging have revealed distinct microglial subpopulations with divergent transcriptional profiles and roles in injury progression versus recovery (Olah et al. 2020; Kim and Jung 2024; Nevelchuk et al. 2024). For instance, recent work identified a reparative microglial subset expressing Spp1 that promotes axonal regeneration in rodent SCI models (Shen et al. 2022). Concurrently, preclinical studies exploring microglial modulation—via CSF1R inhibition, genetic ablation, or pharmacological reprogramming—have yielded conflicting results, emphasizing the need for temporally precise interventions (Bellver‐Landete et al. 2019). Clinically, PET imaging using TSPO ligands has enabled in vivo tracking of microglial activation in SCI patients, yet the translational relevance of these findings remains unclear (Best et al. 2019).

Despite notable advancements, the field confronts substantial obstacles. First, a major issue is the lack of standardized nomenclature for the status of microglia cells, such as the terms “M1 versus M2” and “resting versus activated,” which has resulted in inconsistencies across various studies (Paolicelli et al. 2022). Second, most experimental studies mainly come from rodent models, which may not fully replicate the complex pathophysiology of human SCI (Nardone et al. 2017; Younsi et al. 2021). Third, the complex interactions between microglia and other immune cells, such as infiltrating macrophages and astrocytes, are not fully understood, which hinders the development of treatment (Liddelow et al. 2020; Singh et al. 2011). Therefore, there is an urgent need for a systematic and large‐scale analysis of existing literature to synthesize fragmented knowledge, determine consensus findings, and prioritize translational research approaches.

Bibliometrics, a computational framework combining quantitative analysis and network science, has become indispensable for mapping evolving research landscapes (Ninkov et al. 2022). By analyzing publication trends, citation networks, and keyword clusters, this approach can reveal shifts in scientific focus, evaluate the impact of technological breakthroughs, and uncover understudied areas (Roldan‐Valadez et al. 2019; Azizan 2024c). Bibliometric studies have been applied to broader SCI research, there are studies focusing on the scientific progress of extracellular vesicles in the field of SCI (Zhiguo et al. 2023), as well as on the research hotspots and trends of microRNAs in the field of SCI (B. Hu et al. 2024), none have specifically addressed the microglial dimension—a critical gap given the cell's therapeutic relevance.

With the increasing volume of research literature exploring the relationship between microglia and SCI, there is an urgent need to visually synthesize and analyze this information to offer valuable insights for future medical research. Consequently, this study specifically conducted bibliometric analysis on microglia and established three unique contributions in the field of SCI: (1) It revealed that microglia are at the core of SCI pathology, unlike more extensive studies on glial cells/macrophages; (2) by using three analysis software (VOSviewer, Bibliometrix, and CiteSpace) for bibliometric analysis, the limitations of a single tool in existing literature have been overcome; and (3) it is crucial to link bibliometric trends with therapeutic innovation, providing a direct pathway for targeted clinical strategies that prioritize polarization regulation of microglia.

## 2 Methods

### 2.1 Data Collection

**FIGURE 1:**
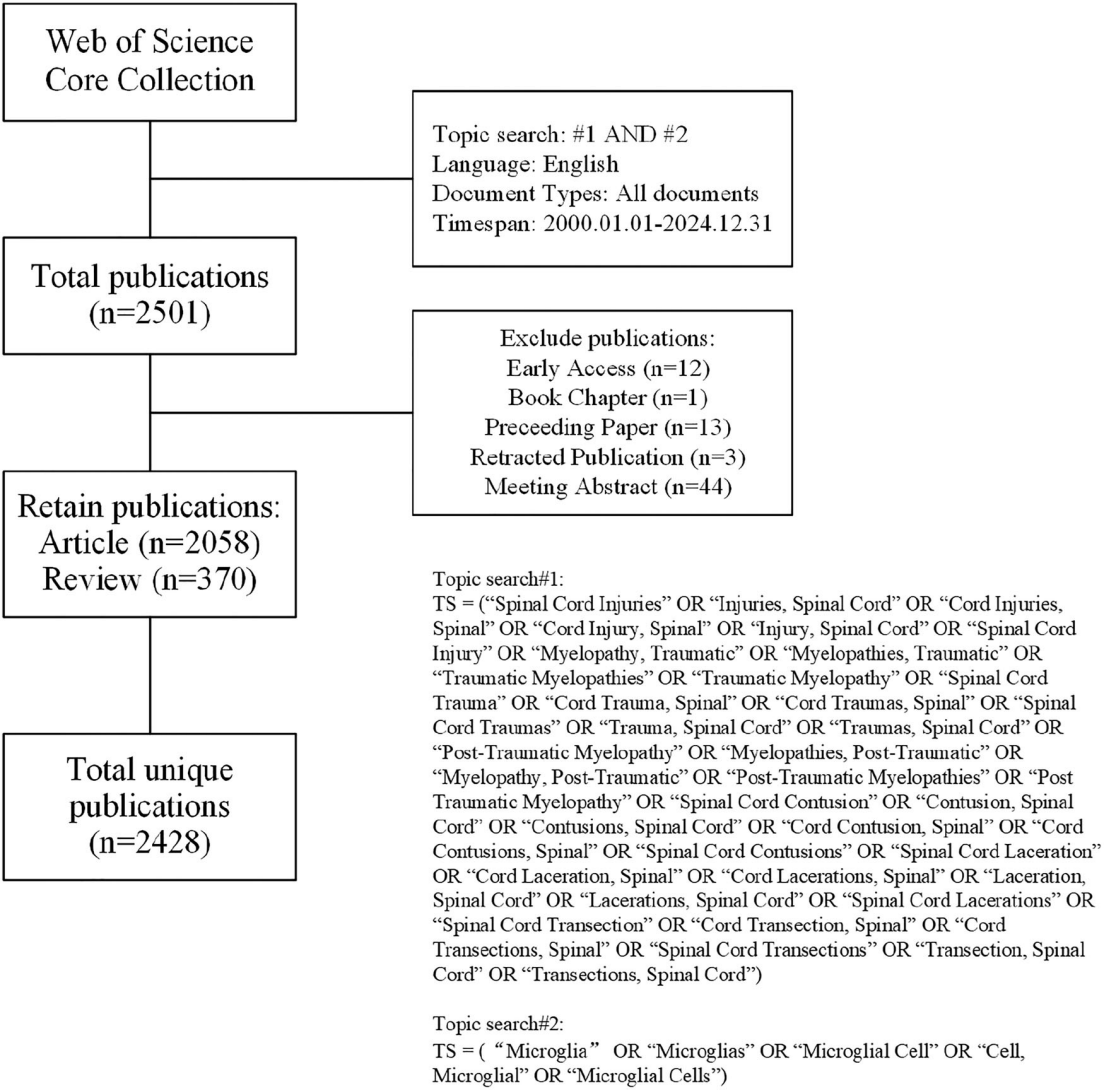
The flowchart of study identification and selection.

The Web of Science Core Collection (WoSCC) functioned as the principal database for data retrieval. As one of the world's largest and most comprehensive online databases, WoSCC provides an extensive collection of scientific studies and analyses that hold significant authority and reference value (Qin et al. 2022). In our study, the retrieval formula is set to: TS = (“Spinal Cord Injuries” OR “Injuries, Spinal Cord” OR “Cord Injuries, Spinal” OR “Cord Injury, Spinal” OR “Injury, Spinal Cord” OR “Spinal Cord Injury” OR “Myelopathy, Traumatic” OR “Myelopathies, Traumatic” OR “Traumatic Myelopathies” OR “Traumatic Myelopathy” OR “Spinal Cord Trauma” OR “Cord Trauma, Spinal” OR “Cord Traumas, Spinal” OR “Spinal Cord Traumas” OR “Trauma, Spinal Cord” OR “Traumas, Spinal Cord” OR “Post‐Traumatic Myelopathy” OR “Myelopathies, Post‐Traumatic” OR “Myelopathy, Post‐Traumatic” OR “Post‐Traumatic Myelopathies” OR “Post Traumatic Myelopathy” OR “Spinal Cord Contusion” OR “Contusion, Spinal Cord” OR “Contusions, Spinal Cord” OR “Cord Contusion, Spinal” OR “Cord Contusions, Spinal” OR “Spinal Cord Contusions” OR “Spinal Cord Laceration” OR “Cord Laceration, Spinal” OR “Cord Lacerations, Spinal” OR “Laceration, Spinal Cord” OR “Lacerations, Spinal Cord” OR “Spinal Cord Lacerations” OR “Spinal Cord Transection” OR “Cord Transection, Spinal” OR “Cord Transections, Spinal” OR “Spinal Cord Transections” OR “Transection, Spinal Cord” OR “Transections, Spinal Cord”) AND TS = (“Microglia” OR “Microglias” OR “Microglial Cell” OR “Cell, Microglial” OR “Microglial Cells”). The search was performed within the time frame of January 1, 2000, to December 31, 2024, to identify relevant articles for inclusion. The search was restricted to English‐language articles, and only “Articles” and “Reviews” were selected as the article types. Articles written in other languages, “Proceeding Paper,” “Early Access,” “Book Chapters,” and “Retracted Publication” were excluded. This study invited two experts with a background in bibliometrics or information retrieval to review the preliminary design of the retrieval strategy, and conducted the search after approval. A total of 2501 articles were retrieved and exported in plain text file format via the Web of Science database. The search results were then data cleaned using CiteSpace software, which exclude articles in duplicate or nonconforming publication formats, such as book chapter and meeting abstract, there are ultimately 2428 articles, comprising 2058 articles and 370 reviews. To avoid potential bias resulting from subsequent database updates, all searches and downloads were completed on a single day, namely, January 21, 2024. The retrieval process is depicted in Figure 1. All articles were exported in TXT format.

### 2.2 Data Analysis

After verifying the exported data, the two authors (Gongpeng Xiong and Ziming Cai) merged redundant elements and corrected spelling errors. The improved dataset was then imported into VOSviewer 1.6.20 and CiteSpace 6.3 R1 for bibliometric analysis. Although VOSviewer and CiteSpace are primarily responsible for visualizing data analysis, the publication and citation trends of literature over the years have been generated using Microsoft Excel 365.

VOSviewer is a robust bibliometric analysis software that enables data extraction and processing (van Eck and Waltman 2010). It is primarily employed for visualizing collaborative networks among countries, institutions, authors, and journals, as well as the co‐citation of keyword clusters. Bibliometrix, developed using the R programming language, is a data visualization tool designed for comprehensive bibliometric mapping analysis via the Biblioshiny application. It summarizes publications and citations by country, institution, journal, and author (F. Wu et al. 2022). Furthermore, CiteSpace is a widely utilized bibliometric analysis software that offers a clear understanding of research hotspots and evolutionary processes in specific fields, thereby providing insights into future development directions (Synnestvedt et al. 2005). CiteSpace is especially adept at analyzing citation bursts and keyword bursts to identify research hotspots. Additionally, it offers other visual analysis functions, such as clustering publication data and generating keyword timeline graphs, among others, to help researchers gain a deeper understanding of the historical and current state of a discipline (Liu et al. 2024). We also referred to relevant methodological benchmarks, which helped to combine content analysis with bibliometric mapping and provided a powerful model for topic analysis and scientific collaboration networks, pointing out future directions for qualitative SCI microglial research (Azizan 2024a, 2024b).

In our study, we conducted bibliometric analysis following the guidelines of Donthu et al. (2021) and developed a five stage bibliometric workflow: First, we defined the scope by focusing on the application of microglia in the field of SCI (2000–2024) and developed a retrieval strategy (Phase 1). Next, an expert‐validated retrieval strategy was executed in WOS (Phase 2). After deduplication and standardization of data (Phase 3), we utilized VOSviewer (version 1.6.20), R software (version 4.1.2), and Bibliometric Online Analysis Platform (https://bibliometric.com/) to analyze country/region distribution, institution distribution, author collaboration and distribution, and keyword distribution and collaboration. Simultaneously, we employed CiteSpace (version 6.3.1) to analyze the dual‐map overlay of journals, reference collaboration and distribution, literature bursts, and keyword bursts (Phase 4). Finally, we explained the results by analyzing clinical issues and emerging research focuses (Phase 5).

## 3 Results

### 3.1 Annual Publications and Citation Trends

**FIGURE 2:**
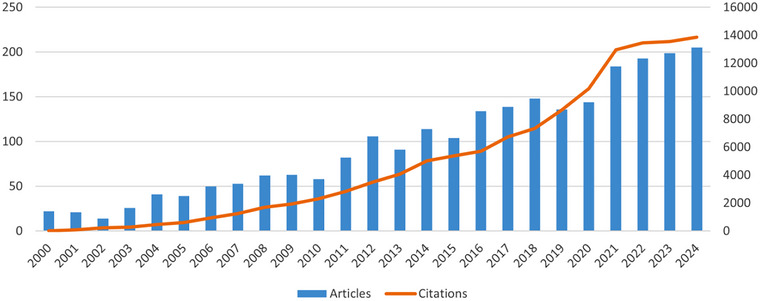
Annual publications and citation trends on research of microglia in spinal cord injury.

To a certain extent, the volume of published literature serves as an indicator of the research level and developmental overview within a field. Figure 2 illustrates the number of publications and their corresponding citation trends from 2000 to 2024. It can be observed that from 2000 to 2020, the number of publications on SCI exhibited a wave‐like growth pattern, with the number of publications surpassing 100 for the first time in 2012. However, from 2014 to 2024, there was a rapid increase in the number of publications, with the most significant surge occurring between 2020 and 2021. Clearly, the frequency of citations has been on the rise year by year. Overall, research on microglia in SCI demonstrates a pronounced trend of rapid development.

### 3.2 Distributions of Countries/Regions

**FIGURE 3:**
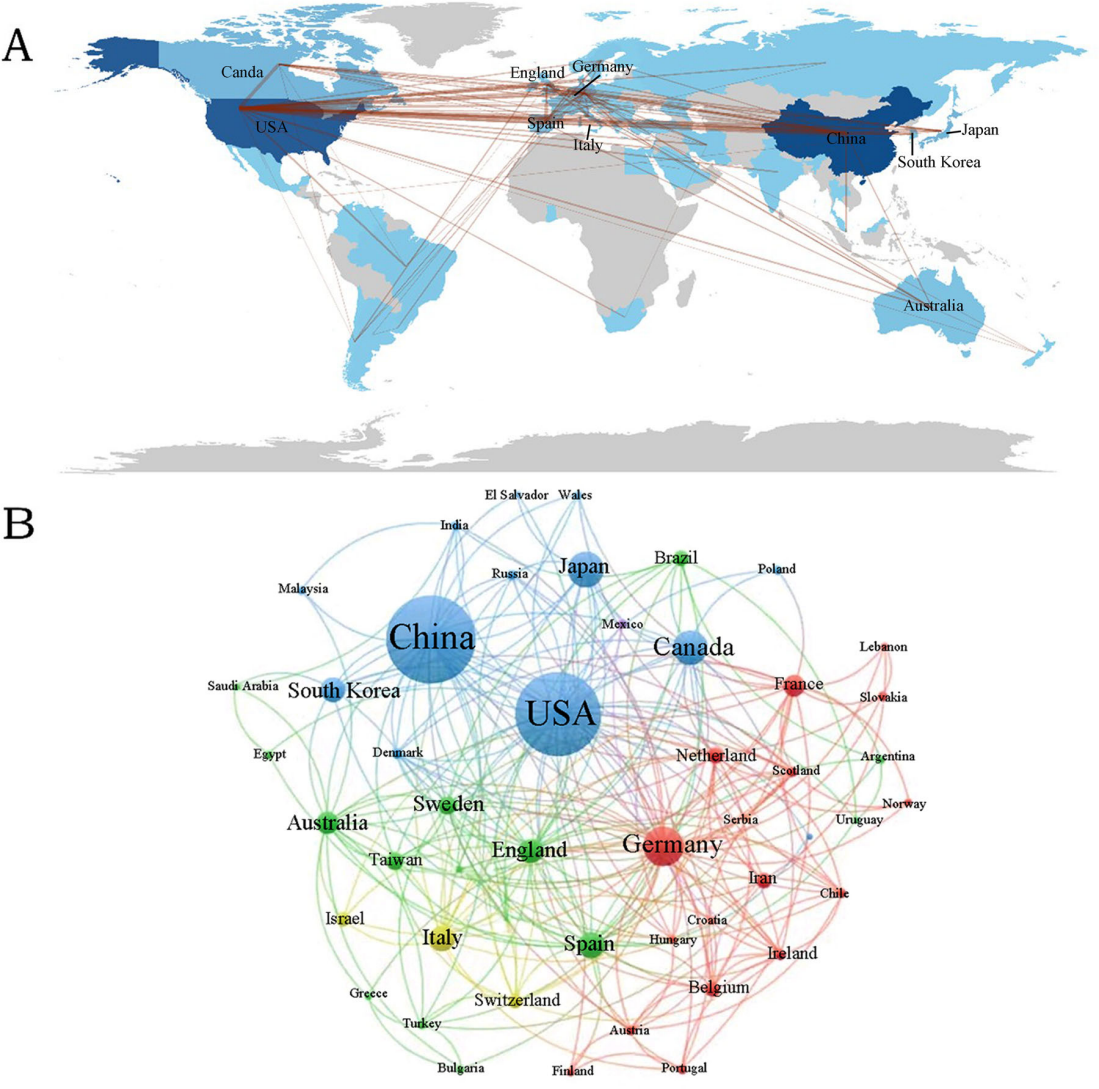
(A) Countries/regions collaboration map. (B) Distributions of countries/regions and collaboration network.

At present, 61 countries or regions are engaged in research on microglia in SCI, with the majority of research activity concentrated in the Northern Hemisphere. Moreover, the connections between these countries and regions are predominantly located in the Northern Hemisphere. By contrast, Australia, Brazil, and Argentina, situated in the Southern Hemisphere, exhibit relatively high levels of activity in this field, maintaining a higher frequency of communication with other countries (Figure 3A). As depicted in Table 1, the top 10 countries or regions with the highest number of published papers in this field are headed by China (773 papers, 35.01%), closely followed by the United States (705 papers, 31.93%). The fact that the United States and China, the two countries with the most publications, together account for more than half of the total indicates that they wield significant influence in this field. Subsequently, Germany (176, 7.97%), Japan (149, 6.75%), and Canada (136, 6.16%) follow closely, all holding important positions in this research field. In terms of overall connection strength, the top five are the United States (337), Germany (159), China (149), England (80), and Canada (77). We also employed VOSviewer to visually analyze countries or regions (Figure 3B). According to the degree of cooperation, the cooperation between countries and regions can be divided into 10 groups. It is evident from the analysis that the level of cooperation between countries and regions is relatively high.

**TABLE 1:** Top 10 most publication countries/regions related to microglia in spinal cord injury.

Rank	Country/region	Total link strength	Count (%)
1	China	149	773 (35.01%)
2	USA	337	705 (31.93%)
3	Germany	159	176 (7.97%)
4	Japan	39	149 (6.75%)
5	Canda	77	136 (6.16%)
6	Spain	61	83 (3.76%)
7	Italy	53	79 (3.58%)
8	South Korea	23	78 (3.53%)
9	England	80	72 (3.26%)
10	Australia	34	63 (2.85%)

### 3.3 Analysis of Affiliations

**FIGURE 4:**
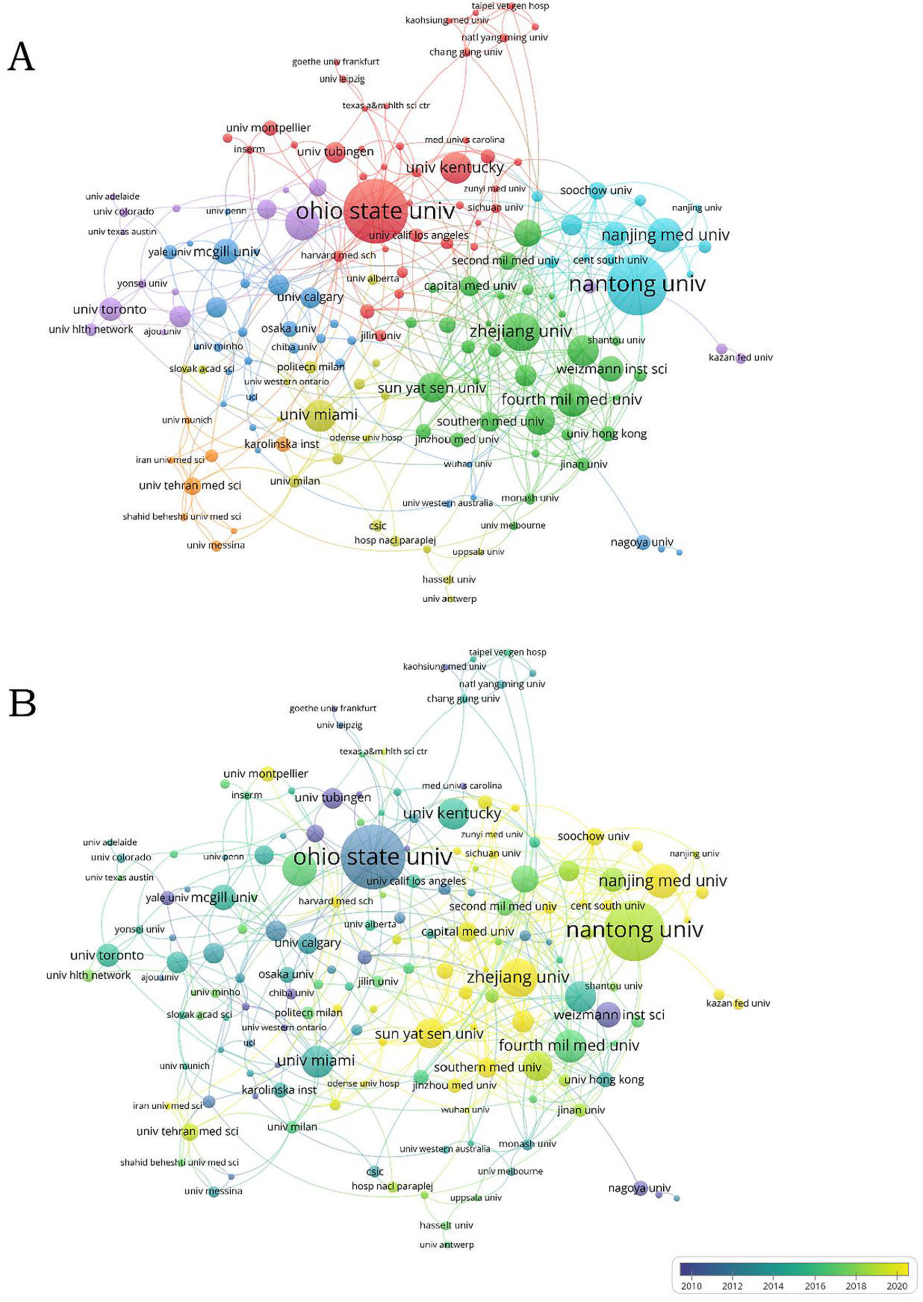
(A) The clustering analysis of the institutions. (B) Network‐view map of institutional collaboration.

A total of 1886 research institutions have published 2428 studies on the role of microglia in SCI. Table 2 presents the top 10 institutions based on the number of published articles. Three institutions have published more than 40 articles. Ohio State University (71, 3.22%) ranks first, followed by Nantong University (67, 3.03%) and Zhejiang University (42, 1.90%). Sun Yat‐Sen University has the strongest comprehensive correlation, followed by Nanjing Medical University and Rogers State University. The majority of the top 10 universities are located in China, highlighting the significant role Chinese universities play in maintaining the country's prominent position in this field. Figure 4A shows the clustering analysis of the mechanism, and the collaborative network is visually analyzed using VOSviewer. It is evident that there has been consistent and close cooperation among these institutions. Ohio State University, Nantong University, and other institutions have progressively developed self‐centered collaborative networks. In Figure 4B, the Weizmann Institute of Science, Tubingen University, Georgetown University, and other institutions are primarily represented in blue and have conducted research in this field earlier. Chinese institutions, including Nantong University, Zhejiang University, and Sun Yat‐Sen University, are mainly represented in green and yellow, indicating that China has entered the field and published a substantial number of articles within a relatively short timeframe in recent years. Ohio State University is depicted in blue with the largest circle node, suggesting that the institution might have been the leading research entity in this field in the past. Nantong University is represented in green with the second largest circle node, suggesting that it might currently be a relatively strong research institution in this field and could potentially spearhead future research. Zhejiang University, Sun Yat‐Sen University, and Nanjing Medical University all have yellow and large circular nodes, indicating that these institutions may become an important force in advancing the development of this field.

**TABLE 2:** Top 10 most publication institutions related to microglia in spinal cord injury.

Rank	Institutions	Total link strength	Count (%)
1	Ohio State University	39	71 (3.22%)
2	Nantong University	32	67 (3.03%)
3	Zhejiang University	33	42 (1.90%)
4	Nanjing Medical University	43	38 (1.72%)
5	University of Maryland	26	38 (1.72%)
6	Fourth Military Medical University	26	36 (1.63%)
7	University of Miami	38	35 (1.63%)
8	Rogers State University	41	34 (1.54%)
9	University of Kentucky	12	34 (1.54%)
10	Sun Yat‐Sen University	53	32 (1.45%)

### 3.4 Contributions of Journals

**FIGURE 5:**
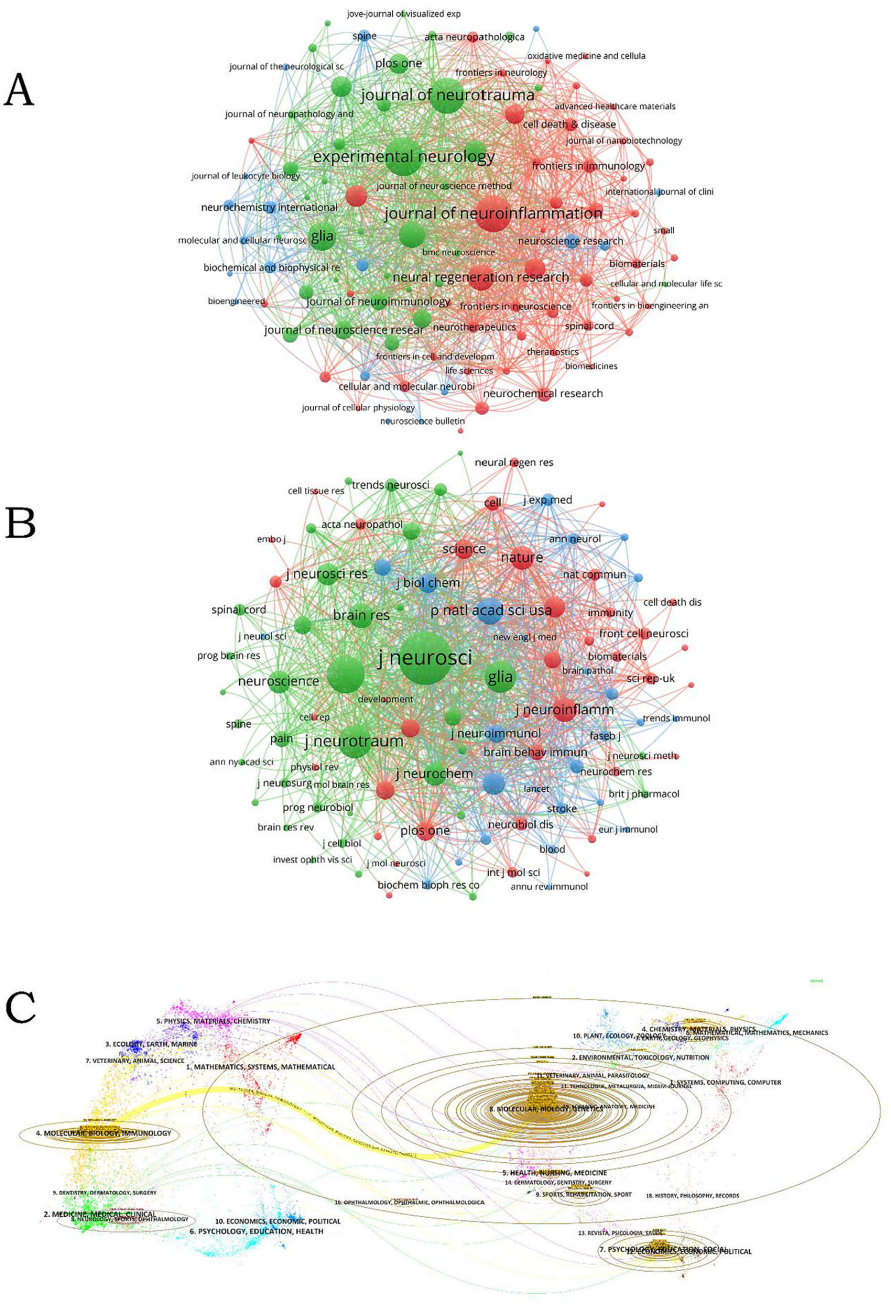
(A) Journals clustering analysis of microglia in spinal cord injury. (B) Co‐cited journals clustering analysis of microglia in spinal cord injury. (C) The dual‐map overlay of journal publishing research.

A total of 2428 articles on microglia in SCI were published across 74 journals. Table 3 indicates that *Experimental Neurology* published the highest number of papers (99, 4.48%), followed by the *Journal of Neuroinflammation* (91, 4.12%) and the *Journal of Neurotrauma* (88, 3.99%). Among the top 10 journals with the highest number of published papers, seven are in Q1 and two are in Q2, with the *Journal of Neuroinflammation* having the highest impact factor (IF) of 9.3. In the top 10 most cited journals, Q1 accounts for seven and Q2 for two, indicating significant influence and high acclaim in this field. This suggests notable research achievements and a relatively high overall research level, with potential for further in‐depth investigation. The impact of a journal largely depends on its citation count, as this reflects the extent to which its articles are referenced and utilized by scholars and researchers. Among the top 10 cited journals, the *Journal of Neuroscience* has the most citations (9031), indicating its important impact on the study of microglia in SCI, followed by *Experimental Neurology* (5037) and the *Journal of Neurotrauma* (4234). Figure 5A,B shows the clustering analysis of journals and co‐cited journals, and VOSviewer software is used to visually analyze the collaboration network between them, providing a clearer view of detailed cooperation. Figure 5A illustrates three distinct thematic clusters: the green cluster represents fundamental mechanisms of neurochemistry and glial cell biology; the blue cluster serves as an interdisciplinary nexus, integrating diverse scientific domains; while the red cluster embodies translational medicine and clinical applications, with a specific focus on neural injury, repair, and regeneration. In Figure 5B, the green cluster reflects the core themes of basic neuroscience research, the blue cluster denotes applied neuroscience and emerging interdisciplinary frontiers, and the red cluster comprises the world's leading multidisciplinary scientific journals and top‐tier life science publications. Articles in the same journal may share similar research directions or internal logic. It is evident that *Experimental Neurology*, the *Journal of Neurotrauma*, and *Glia* have higher co‐citation rates and greater impact. In the dual‐map overlay of journal‐published research (Figure 5C), a prominent cited pathway, denoted by yellow, is observed. The colored path between the left and right sides illustrates the citation relationships between various fields, revealing that studies published in journals focused on molecular, biological, and genetic research primarily cite research published in journals specializing in molecular, biological, and immunological research. This indicates the interdependence and interconnectedness of various fields, contributing to our understanding of SCI.

**TABLE 3:** Top 10 journals and co‐cited journals related to microglia in spinal cord injury.

Rank	Journal	Count (%)	IF (2024)	JCR quartile	Co‐cited journal	Citation	IF (2024)	JCR quartile
1	*Experimental Neurology*	99 (4.48%)	4.2	Q1	*Journal of Neuroscience*	9031	4.0	Q2
2	*Journal of Neuroinflammation*	91 (4.12%)	10.1	Q1	*Experimental Neurology*	5037	4.2	Q1
3	*Journal of Neurotrauma*	88 (3.99%)	3.8	Q1	*Journal of Neurotrauma*	4234	3.8	Q1
4	*Glia*	61 (2.76%)	5.1	Q1	*Glia*	3878	5.1	Q1
5	*Journal of Neuroscience*	55 (2.49%)	4.0	Q2	*Proceedings of the National Academy of Sciences of the United States of America*	2908	9.1	Q1
6	*Neural Regeneration Research*	52 (2.36%)	6.7	Q1	*Journal of Neuroinflammation*	2547	10.1	Q1
7	*Brain Research*	41 (1.86%)	2.6	Q3	*Brain Research*	2500	2.6	Q3
8	*Brain Behavior and Immunity*	40 (1.81%)	7.6	Q1	*Nature*	2492	48.5	Q1
9	*Frontiers in Cellular Neuroscience*	38 (1.72%)	4.0	Q2	*Journal of Neurochemistry*	2266	4.0	Q2
10	*Neuroscience*	38 (1.72%)	2.8	Q3	*Neuroscience*	2245	2.8	Q3

### 3.5 Authors and Co‑Cited Authors

**FIGURE 6:**
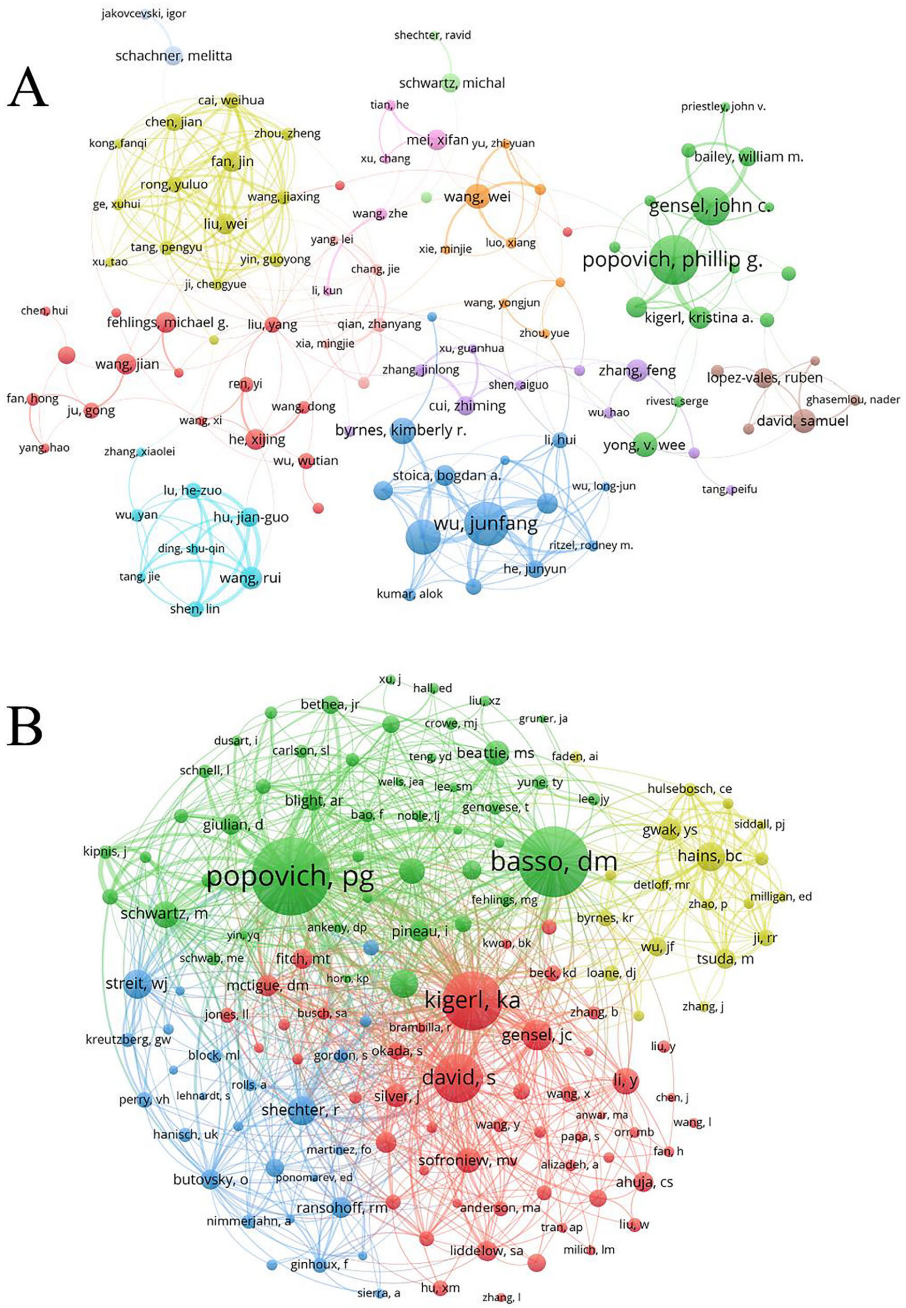
(A) Coauthor related to microglia in spinal cord injury. (B) Network of co‐cited authors. The size of the node depends on the author's co‐citation.

A total of 11,069 authors have contributed to publications related to microglia in SCI. Table 4 presents the top 10 authors based on the number of publications and citation frequency, highlighting key researchers and their contributions to advancing our understanding of microglia in SCI. Table 5 lists the top 10 authors based on the *H*‐index and *G*‐index, which can be used to evaluate the quantity and level of academic output of researchers. The *H*‐index measures the balance between the quality and quantity of academic output of scholars, while the *G*‐index emphasizes the cumulative impact of highly cited achievements. Popovich, Pg has the highest number of publications in this field, with 33 papers (1.49% of all publications), followed by Wu, Junfang (29, 1.31%) and Gensel, Jc (25, 1.31%). Wu, Junfang and Faden, Ai exhibit higher total link strength, indicating extensive collaborations with other researchers. Among the top 10 co‐cited authors, Popovich, Pg (728 citations), Basso, Dm (653 citations), and Kigerl, Ka (537 citations) have been cited more than 500 times, while eight authors have been cited more than 250 times, signifying their pivotal roles in the field. The collaborative network of authors in the literature on microglia in SCI was visualized using VOSviewer, with different colors representing distinct clusters. Coauthors formed 12 clusters (Figure 6A), among which the red, dark blue, and yellow groups have more extensive connections with external researchers. Figure 6B displays the collaboration network of co‐cited authors, partitioned into three distinct clusters. Only the blue group (Chong Zz, Maiese K) had sparse connections with the other two groups. Heneka Mt has the highest number of co‐citations, followed by Popovich, Pg and Basso, Dm, who are representative figures and core research forces in the field, enjoying high academic prestige.

**TABLE 4:** Top 10 authors and co‐cited authors related to microglia in spinal cord injury.

Rank	Author	Count (%)	Total link strength	Co‐cited author	Citation	Total link strength
1	Popovich, Pg	33 (1.49%)	64	Popovich, Pg	728	24,032
2	Wu, Junfang	29 (1.31%)	107	Basso, Dm	653	13,627
3	Gensel, Jc	25 (1.13%)	42	Kigerl, Ka	537	18,307
4	Faden, Ai	23 (1.04%)	71	David, S	445	13,353
5	Byrnes, Kr	17 (0.77%)	22	Gensel, Jc	270	9037
6	Wang, Wei	16 (0.72%)	33	Shechter, R	253	9742
7	Yong, Vw	16 (0.72%)	15	Hains, Bc	252	8477
8	David, S	15 (0.68%)	24	Streit, Wj	252	7489
9	Wang, Rui	14 (0.63%)	50	Donnelly, Dj	244	7968
10	Kigerl, Ka	14 (0.63%)	36	Schwartz, M	234	8152

**TABLE 5:** Top 10 authors' *H*‐index and *G*‐index related to microglia in spinal cord injury.

Rank	Author	*H*‐index	Author	*G*‐index
1	Popovich, Pg	30	Popovich, Pg	32
2	Wu, Junfang	27	Wu, Junfang	29
3	Gensel, Jc	23	Gensel, Jc	24
4	Faden, Ai	21	Faden, Ai	23
5	Byrnes, Kr	17	Byrnes, Kr	21
6	Yong, Vw	15	Wang, Wei	16
7	Wang, Wei	14	Yong, Vw	16
8	David, S	14	Chen, J	13
9	Wang, Rui	13	Wang, J	13
10	Kigerl, Ka	11	Zhang, Y	12

### 3.6 References and Co‐Cited References

Table S1 displays the 15 most frequently cited articles among the 2428 retrieved papers. “Identification of two distinct macrophage subsets with divergent effects causing either neurotoxicity or regeneration in the injured mouse spinal cord” is the most cited article (3439 citations) (Kigerl et al. 2009). This article aims to explore in depth the mechanism of macrophages derived from microglia in the central nervous system during secondary injury and repair after SCI. This article also deeply analyzes the phenotypic differentiation of microglia and macrophages after SCI and their effects on the pathological process and repair ability of SCI, providing new ideas and directions for the treatment of SCI. The second most cited article is “Neuroinflammation and M2 microglia: the good, the bad, and the inflamed” with 1204 citations (Cherry et al. 2014). This article aims to explore the potential role of M2 microglia in the process of neuroinflammation in SCI and other different neurological diseases, with a focus on analyzing the activation mechanism, functional characteristics, and impact on neuroprotection, inflammation regulation, and disease progression of M2 microglia. The authors of the article, “Repertoire of microglial and macrophage responses after spinal cord injury,” which has the third highest number of citations at 1059, offer a synopsis of the role of neuroinflammation in SCI (David and Kroner 2011). This article aims to explore the response of microglia after SCI and its role in different pathological environments. By elaborating on the response mechanism of microglia after SCI and its impact on injury recovery, it provides a new perspective for understanding the pathophysiological process of SCI and an important reference for future research on new treatment methods for SCI. “Neuroinflammation: the devil is in the details,” the fourth most cited article with 883 citations, aims to explore in depth the complex roles in the central nervous system and their manifestations under different diseases and injury conditions, with a particular focus on SCI and the role of microglia in this process (DiSabato et al. 2016). The article emphasizes the central position of microglia, the dual nature of neuroinflammation and its regulatory mechanisms, as well as the complexity of neuroinflammation in SCI and the core role of microglia in this process, which may provide new strategies for the treatment of neurological diseases such as SCI.

**FIGURE 7:**
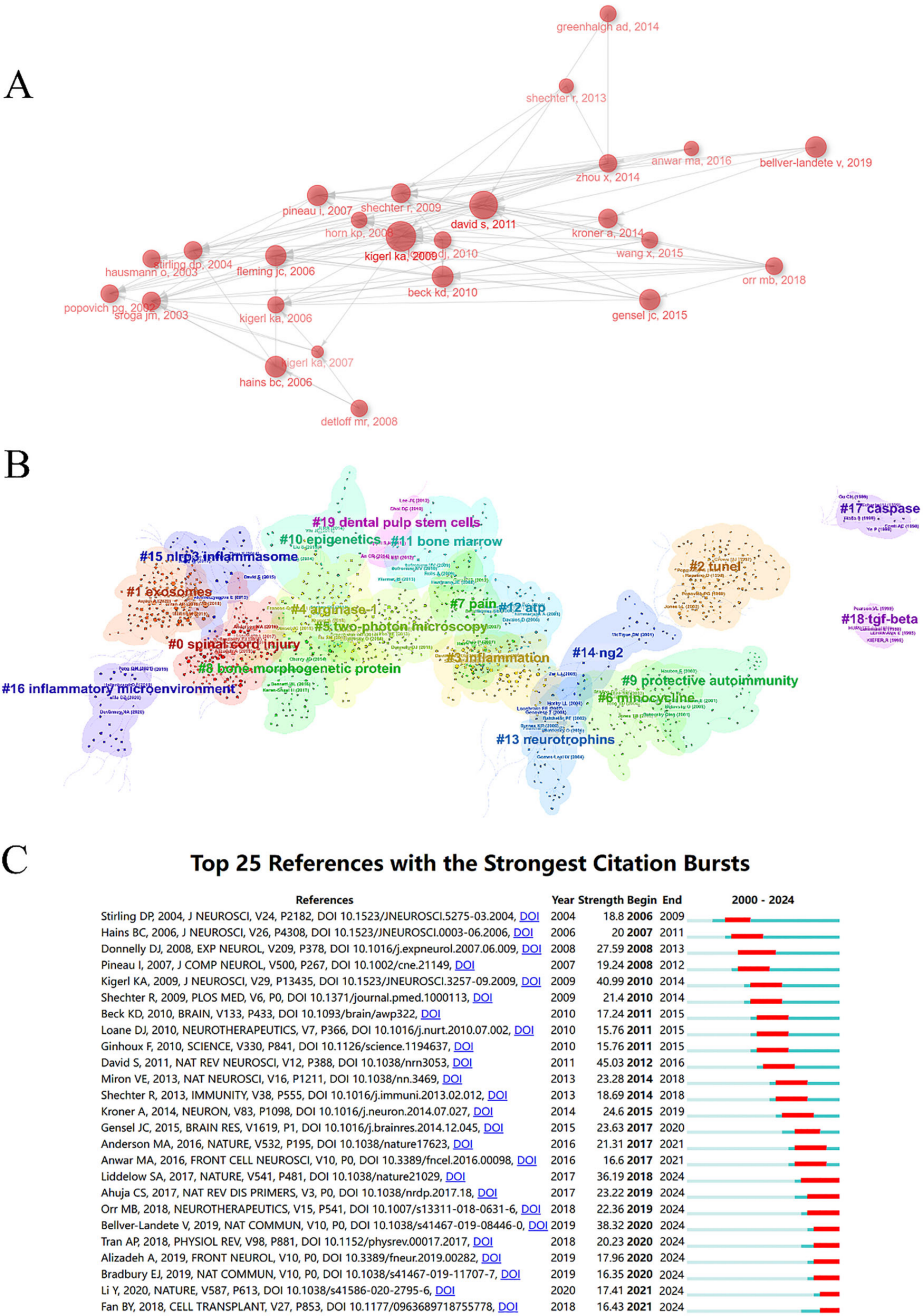
(A) The network diagram of document relationship obtained by VOSviewer. (B) Cluster view of references on microglia in spinal cord injury. (C) CiteSpace visualization map of top 25 references with the strongest citation bursts involved in microglia in spinal cord injury.

Through co‐citation analysis of literature references, we constructed a network of cited documents, and through the arrows between the various graphics, we can see the relationships between the cited literature (Figure 7A) and performed cluster analysis using CiteSpace, which generated 15 distinct co‐citation clusters (Figure 7B). A temporal analysis revealed a publication timeframe (2006, 2009, and 2011) exhibiting explosive citation bursts, with these years containing the highest concentration of highly cited studies. Notably, articles demonstrating substantial citation frequency formed the core of these burst clusters, indicating a strong correlation between research impact (as measured by citation bursts) and scholarly attention (Yang et al. 2024). As depicted in Figure 7B, the cluster diagram demonstrates a robust modular structure with a modularity *Q* value of 0.7811 and a weighted mean silhouette value of 0.9284, indicating high stability and analytical validity. Cluster #0 represents the largest aggregation, with its size reflecting the citation impact of associated publications. The most frequent keywords across clusters are hierarchically distributed: “spinal cord injury” predominates, followed sequentially by “exosomes” (Cluster #1), “TUNEL” (Cluster #2), “inflammation” (Cluster #3), and “arginase‐1” (Cluster #4). Additional significant clusters include “two‐photon microscopy,” “minocycline,” and “pain.” Temporal analysis through Figure 7A reveals emerging research priorities in recent years, particularly “exosomes,” “microglia,” and “inflammatory microenvironment.” Figure 7C identifies the 25 most citation‐burst references in SCI microglia research, providing chronological insights into evolving research trends. The seminal review “Repertoire of microglial and macrophage responses after spinal cord injury” (David and Kroner 2011) exhibits the strongest citation burst (strength = 45.03) with sustained impact from 2012 to 2016. This bibliometric profile systematically maps disciplinary evolution and highlights pivotal knowledge transitions in the field (David and Kroner 2011).

### 3.7 The Analysis of Hotspots and Frontiers

#### 3.7.1 Keywords Co‐Occurrence and Cluster Analysis

**FIGURE 8:**
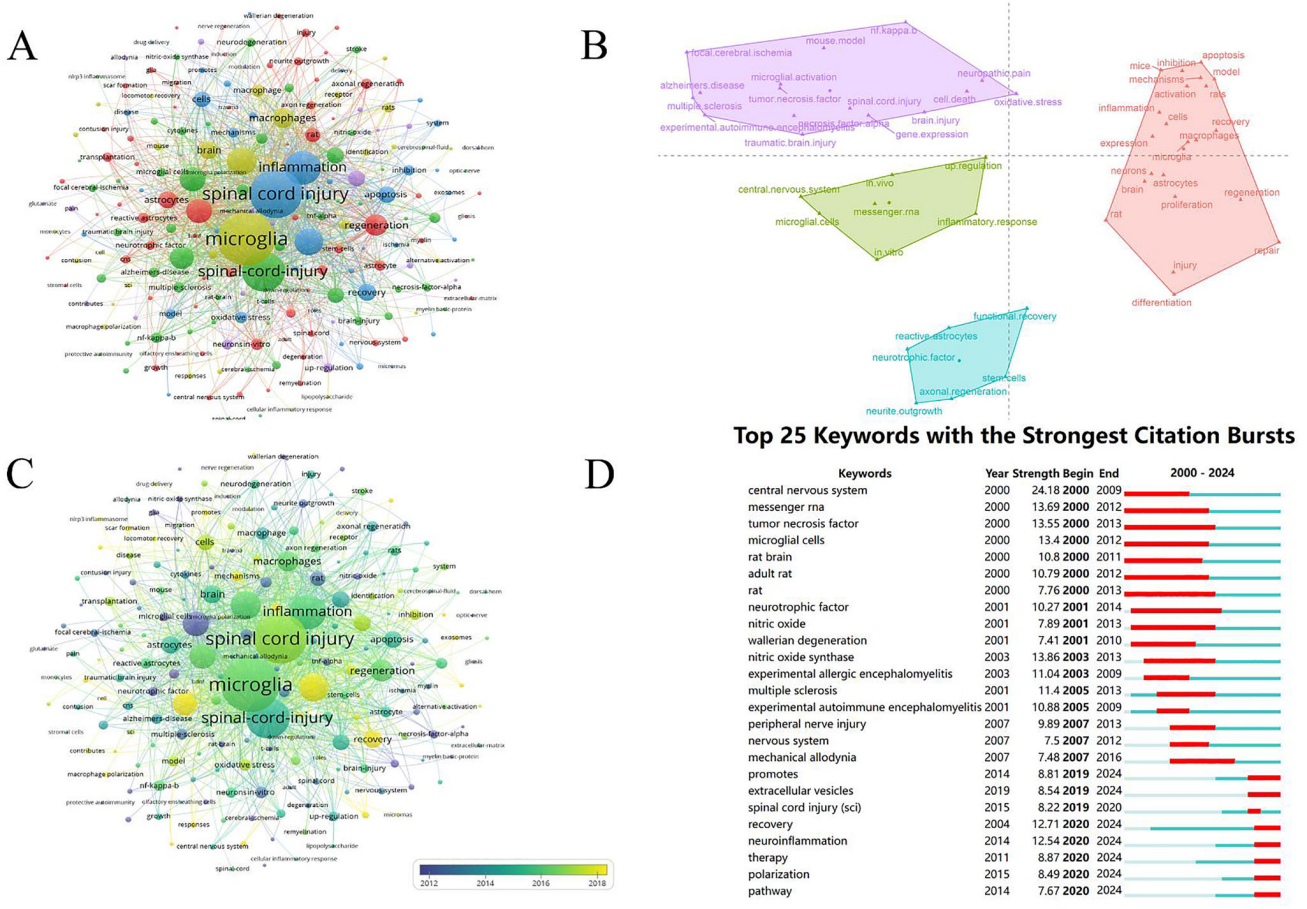
(A) The keyword co‐occurrence network relationship clustering diagram. (B) A map of the conceptual structure of the top 50 terms in terms of word frequency. (C) Network‐view map of keyword co‐occurrence. (D) CiteSpace visualization map of top 25 keywords with the strongest citation bursts.

The keyword clustering results are presented in Figure 8A. Four distinct clusters were identified through analysis: the blue cluster primarily centered on “spinal cord injury” and “inflammation,” while the green cluster encompassed key terms such as “central nervous system” and “neuroinflammation.” The yellow cluster featured prominent keywords including “microglia” and “brain,” whereas the red cluster was characterized by terms like “regeneration” and “astrocytes.” This clustering pattern effectively reveals current research priorities and emerging frontiers, providing valuable insights to guide future investigations in the field (Çoşkun and Metin 2025). This analysis elucidates prominent and emerging research domains in SCI, particularly highlighting the functional roles of microglia and astrocytes in SCI pathogenesis. The findings further demonstrate that disease progression is crucially linked to neuroinflammatory processes and cytokine‐mediated pathways. These insights collectively delineate critical mechanistic frameworks, offering critical guidance for prioritizing research directions in SCI investigation.

Figure 8B presents the results of multiple correspondence analysis (MCA) generated using the MCA function from the FactoMineR package. The *x* and *y* axes represent Dim1 and Dim2 derived from the MCA, with values in parentheses indicating the cumulative percentage of variance explained by each dimension. This figure integrates clustering analysis with MCA visualization of keywords. The graphical representation of keyword MCA allows qualitative assessment of their similarity based on spatial proximity in the coordinate system, while the clustering algorithm enhances the interpretability of these similarity relationships through explicit grouping patterns (Seredin et al. 2024). As illustrated in Figure 8B, the top 50 ranked keywords are classified into four distinct clusters, with higher similarity among keywords within the same cluster potentially representing specific thematic domains. The purple cluster focuses on specific neurological disorders, particularly those resulting from ischemic, inflammatory, or degenerative pathologies. The green cluster represents the most fundamental and central research direction, concentrating directly on microglia—the key cellular components within the central nervous system. The red cluster broadens the perspective beyond the central nervous system to encompass cellular mechanisms of immune response and tissue repair. Lastly, the blue cluster embodies therapeutic strategies and regenerative potential, emphasizing intrinsic factors that promote neural regeneration and repair.

#### 3.7.2 Keywords Timeline Viewer

Figure 8C illustrates the temporal evolution of keywords through a color gradient (blue to red) representing their mean publication years. High‐frequency keywords, including spinal cord injury (mean year: 2016), inflammation, regeneration, microglia, macrophages, and astrocytes, predominantly emerged between 2015 and 2017. A chronological progression is evident: microglia polarization and neurons in vitro (2012); spinal cord injury, microglia, and inflammation (2015); followed by neuroinflammation, activation, recovery, and mechanisms (2017–2018). Recent trends (past 2 years) feature emerging terms like exosomes, extracellular vesicles, and nanoparticles. This temporal pattern highlights a paradigm shift in research priorities, delineating historical trajectories and forecasting future frontiers in the field.

#### 3.7.3 Burst Detection

The keywords, meticulously selected through systematic extraction processes, serve as concise indicators of a paper's core themes. High‐frequency keywords within a research domain effectively reveal its predominant academic focus areas and emerging trends, functioning as quantitative markers of scholarly attention (Sarikhani et al. 2025). Table 6 presents the 20 most frequent keywords identified in SCI microglia research. The top five keywords are “spinal cord injury” (1648 occurrences), “microglia” (1084), “inflammation” (562), “expression” (417), and “activation” (398), with their prevalence indicating current research priorities. Burst detection analysis (Figure 8D) identifies the 25 keywords exhibiting the strongest citation bursts, including “promotes,” “extracellular vesicles,” “recovery,” “neuroinflammation,” “therapy,” “polarization,” and “pathway.” Notably, these burst terms maintain active citation trajectories through 2024, demonstrating their sustained prominence as frontier research directions. This pattern quantitatively confirms these thematic areas as persistent investigative focal points within the discipline.

**TABLE 6:** Top 20 keywords related to microglia in spinal cord injury.

Rank	Keyword	Occurrences	Total link strength	Rank	Keyword	Occurrences	Total link strength
1	Spinal cord injury	1648	15,094	11	Brain	219	2034
2	Microglia	1084	10,252	12	Astrocytes	205	2036
3	Inflammation	562	5487	13	Recovery	204	1833
4	Expression	417	3846	14	Apoptosis	196	1839
5	Activation	398	3761	15	Neuroprotection	191	1902
6	Central nervous system	345	3295	16	Neuropathic pain	186	1677
7	Functional recovery	339	3305	17	Cells	179	1621
8	Neuroinflammation	306	2965	18	Inflammatory response	177	1768
9	Regeneration	291	2845	19	Rat	162	1580
10	Macrophages	257	2549	20	Macrophage	131	1424

#### 3.7.4 Thematic Evolution

**FIGURE 9:**
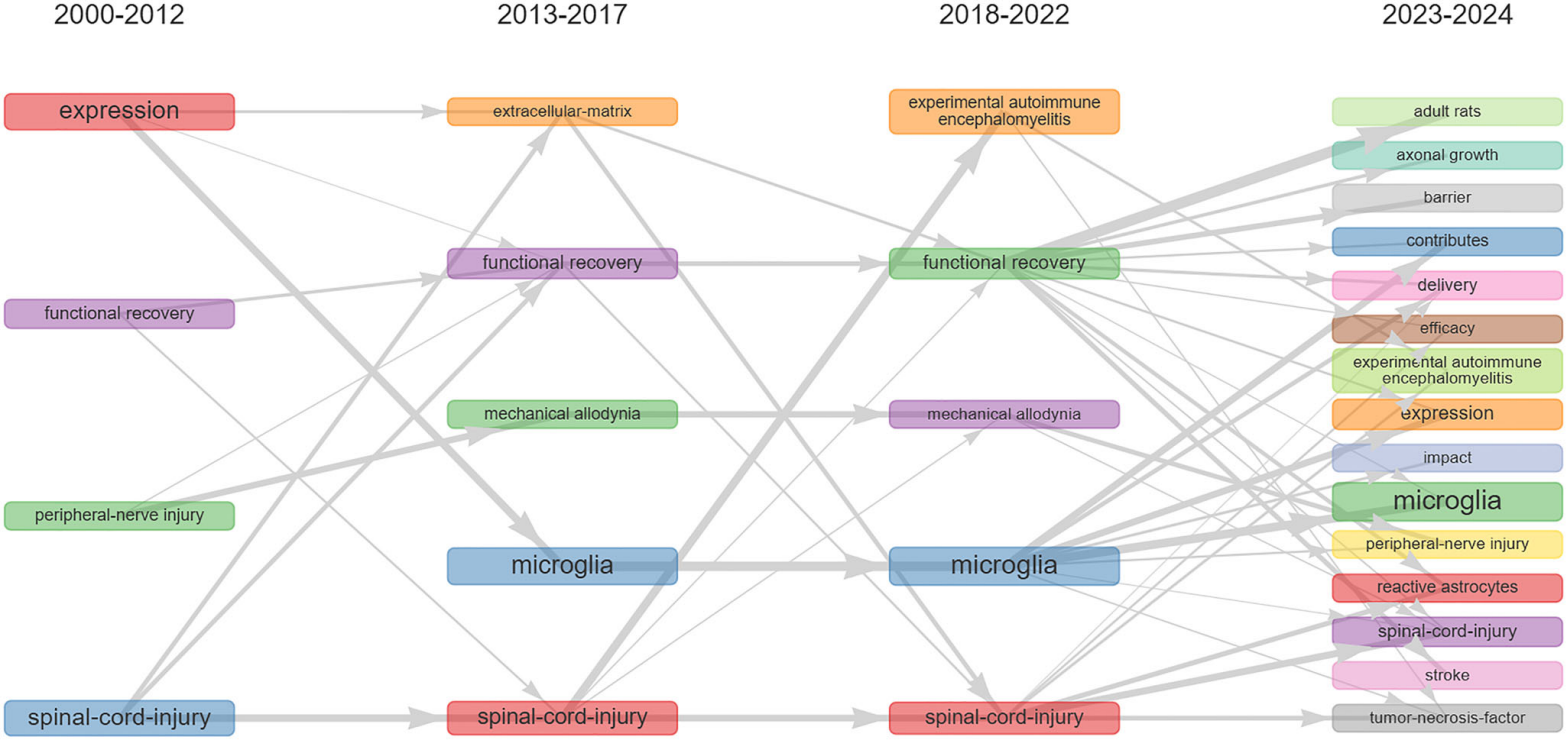
The thematic evolution maps related to microglia in spinal cord injury.

Figure 9 shows the thematic evolution in the field of microglia in SCI research: Early (2000–2012) research focused on basic mechanisms such as gene expression; in the midterm (2013–2017), there was a shift toward functional recovery and regenerative medicine such as extracellular matrix; recently (2018–2022), we have delved into the immune regulation of microglia and the central nervous system; the latest stage (2023–2024) extends to peripheral nerve injury, forming a linkage study between central and peripheral nerves. The theme of “spinal cord injury” that runs through the entire process and the continuously strengthened research on “microglia” reveal the evolution of the field from basic mechanism analysis to treatment strategy expansion. The related themes connected by gray arrows highlight the inheritance and cross development trends of research hotspots.

## 4 Discussion

This study systematically analyzed research foci and evolutionary trajectories in microglia studies related to SCI. A dataset of 2428 Web of Science–indexed publications spanning 2000–2024 was analyzed through integrated bibliometric approaches employing VOSviewer (v1.6.20), CiteSpace (v6.3.1), R (v4.1.2), and the Bibliometric Online Analysis Platform.

### 4.1 General Information

The annual volume and temporal trends of scholarly literature may elucidate the evolutionary trajectory and research progression within a scientific discipline (X. Li et al. 2025). As illustrated in Figure 2, annual publications remained below 50 between 2000 and 2005, reflecting the nascent stage of microglia research during this period. A sustained elevation above 50 publications annually from 2006 to 2010 indicates emerging scholarly engagement with this field. The marked escalation in SCI‐related microglia studies from 2011 to 2024 reveals this subfield's growing scientific significance. Notably, 55.52% of total publications were concentrated in the recent 7‐year span (2017–2024), evidencing intensified academic focus on microglia in SCI research during contemporary years.

Analysis of country/region distribution reveals that China leads in publication volume, while the United States demonstrates superior citation impact and collaborative link strength, ranking second in publication quantity. Notably, China and the United States collectively dominate 66.94% of total publications (China: 35.01%, the United States: 31.93%), establishing them as pivotal contributors to microglia research in SCI, with the United States exhibiting greater research depth and China rapidly narrowing this gap. Institutionally, five of the top 10 organizations by publication output originate from China, whereas five of the top 10 institutions by citation frequency are US based. These metrics underscore the decisive roles both nations play in advancing this field.

As shown in Table 3, *Experimental Neurology* published the most studies on microglia in SCI and was also ranked second among the most cited journal. Seven of the top 10 journals with the most publications are also among the top 10 co‐cited journals (*Experimental Neurology*, *Journal of Neuroinflammation*, *Journal of Neurotrauma*, *Glia*, *Journal of Neuroscience*, *Brain Research*, *Neuroscience*), demonstrating their greater significance in the area. Furthermore, it is worth mentioning that all the top 10 journals are Q1 or Q2, which indicates that the quality of publications in this area is generally high. The top 10 co‐cited journals predominantly cluster within neurosciences, while others exhibit associations with biochemistry, molecular biology, and interdisciplinary domains based on co‐citation frequency analysis, demonstrating consistency with dual‐map overlay trajectories.

In accordance with Table 4, Popovich, Pg has the most papers (33, 1.49%) and citations (728 citations), indicating his greatest influence and most outstanding contributions to the field of microglia in SCI. Popovich, Pg, a professor and chair of Belford Center for Spinal Cord Injury in the Ohio State University, specializes in SCI, brain and spinal cord repair, and molecular biology. In 2009, Popovich et al. published a paper in *Journal of Neuroscience*, titled “Identification of two distinct macrophage subsets with divergent effects causing either neurotoxicity or regeneration in the injured mouse spinal cord” (Kigerl et al. 2009), which discussed the mechanism of macrophages derived from microglia in the central nervous system during secondary injury and repair processes after SCI. This article ranks second among the top 25 cited references in terms of citation strength and first among the top 15 cited references. In 2014, Popovich and others published an article titled “Pattern recognition receptors and central nervous system repair” (Kigerl et al. 2014), which discussed how various PRR families are activated and how they affect the damage and repair process of the central nervous system after injury. The widespread expression of these PRRs in the central nervous system and the release of DAMPs at and around the site of injury indicate that these receptor families play an important role in mediating post injury inflammation. This article ranks among the top 25 in terms of citation strength. In 2020, Popovich and others published an article titled “Microglia organized scar free spinal cord repair in neonatal mice” (Y. Li et al. 2020) in *Nature*, which found that in neonatal mice, scar free healing after SCI was achieved by microglia tissue. Transplanting newly formed microglia or adult microglia treated with peptidase inhibitors into adult mice after injury can improve healing and axonal regeneration. This reveals the cellular and molecular basis of almost complete recovery after SCI in neonatal mice, and proposes strategies that can be used to promote scar free healing of the nervous system in adult mammals. This article has the highest IF in the author's history.

### 4.2 Hotspots and Frontiers

As they represent the main research subjects of a particular field, keywords and references are crucial components of scholarly literature (Pottier et al. 2024). Reference clusters and citation bursts can provide insights into newly popular subjects within the discipline (Hou et al. 2025). Among the top 10 co‐cited references, there are two articles related to the activation status of microglia. One of them explored the role and therapeutic potential of these activated states in neuroinflammation and neurodegenerative diseases (Cherry et al. 2014). Another article explored the fact that after SCI, microglia and newly recruited monocytes differentiate into pro‐inflammatory M1 macrophages, which remain at the site of injury for several weeks, while anti‐inflammatory M2 macrophages have a smaller and shorter response (Kigerl et al. 2009). A study found that in multiple sclerosis (MS), microglia are activated by fibrin that leaks into the central nervous system and binds to CD11b/CD18 receptors, triggering an inflammatory response and exacerbating demyelinating lesions (Davalos and Akassoglou 2012). A study found that microglia are innate immune cells of the CNS, which recognize pathogens and injury signals through pattern recognition receptors such as TLRs and NLRs, activate inflammatory responses, and interact with T cells, affecting the development of neuroinflammation and neurodegenerative diseases (Ransohoff and Brown 2012). A study found that the activation state of microglia has a significant impact on the differentiation direction of neural stem cells. Microglia activated by IL‐4 or IFN‐γ can induce the differentiation of NPCs into neurons and oligodendrocytes, while microglia activated by endotoxins inhibit this differentiation (Butovsky et al. 2006). A study has found a pathway by which microbial metabolites limit the pathogenic activity of microglia and astrocytes and inhibit CNS inflammation (Rothhammer et al. 2018). A study found that the activation and function of microglia play a key role in chronic pain mediated by neuroinflammation, by releasing inflammatory mediators that affect neuronal activity and exacerbate pain (Ji et al. 2018). A study found that microglia are activated in neuroinflammation, producing cytokines and chemokines that affect neuronal function, but excessive activation may lead to neurobehavioral complications (DiSabato et al. 2016). A study has shown that microglia are resident macrophages in the central nervous system, playing important immune surveillance and defense functions in the CNS (David and Kroner 2011). A study has discovered that microglia participate in neuroinflammatory responses in neurodegenerative diseases by releasing pro‐inflammatory cytokines, which have both neuroprotective effects and may lead to neuronal damage and disease progression (Smith et al. 2012).

Moreover, according to Figure 7C, nine references are still in burst and worthy of our attention. Four of the studies focused on how microglia affect scar formation and inflammation in SCI (Bellver‐Landete et al. 2019; Y. Li et al. 2020; Orr and Gensel 2018; Bradbury and Burnside 2019), while the other four explored new treatment pathways and mechanisms after SCI (Ahuja et al. 2017; Tran et al. 2018; Alizadeh et al. 2019; B. Fan et al. 2018), and one study found that neuronal reactive astrocytes are induced by activated microglia (Liddelow et al. 2017). Furthermore, as pictured in Figure 7B, the earliest and largest cluster is #0 (SCI). It is notable that several topics have garnered continuous attention in recent years, including #1(exosomes), #4(arginase‐1), #12 (mitochondria), #14 (ng2), and #15(NLRP3 inflammasome), which are all related to the pathologic mechanism of SCI. Their prominence reflects a paradigm shift toward targeting neuroimmune crosstalk and precision delivery in SCI therapeutics.

As presented in Table 6, the keywords with high occurrence frequencies, aside from the search terms “spinal cord injury” and “microglia,” that include neuroinflammation are “inflammation” (562), “expression” (417), “activation” (398), “central nervous system” (345), and “functional recovery” (339). High‐frequency keywords indicate plenty of popular research directions, including inflammation (inflammation, neuroinflammation, and inflammatory response), cytology (microglia, macrophages, and astrocytes), and neuroscience (central nervous system, neuroprotection, and neuropathic pain). Based on Figure 8C, “exosomes,” “extracellular vesicles,” “nanoparticles,” “NLRP3 inflammasome,” “stem‐cells,” “drug delivery,” and “microRNAs” are hot words in the past two years, representing the frontiers and future development direction. These keywords are mainly related to the pathogenesis and treatment of SCI.

### 4.3 Prospects of Microglia Therapy for SCI

Based on emerging keywords, chronological trends, reference clustering, and high‐intensity outbreak keywords such as “promotion,” “recovery,” “neuroinflammation,” “therapy,” and “polarization,” it is evident that regulating neuroinflammation through the modulation of microglial polarization represents a pivotal and cutting‐edge research focus in the quest to treat SCI effectively.

Microglia, the resident immune cells of the CNS, play a multifaceted role at every stage of SCI, from its onset and progression to tissue repair and recovery (Brennan et al. 2022). These highly plastic cells undergo polarization in response to environmental stimuli, oscillating between two predominant states: the M1 pro‐inflammatory phenotype and the M2 anti‐inflammatory phenotype. The M1 state is primarily involved in initiating and perpetuating the inflammatory response, which, while integral to clearing debris and pathogens, often exacerbates secondary tissue damage. Conversely, the M2 state promotes anti‐inflammatory activity, facilitates tissue repair, and supports neuroprotection. Thus, therapeutic strategies aimed at suppressing M1 polarization while enhancing M2 polarization have garnered significant attention as potential interventions to mitigate neuroinflammation and promote functional recovery in SCI.

One prominent avenue for advancing therapeutic approaches in this context involves the use of exosomes, gut microbiota modulation, and nanoparticles—innovative and rapidly evolving areas of research. Exosomes, nano‐sized extracellular vesicles encapsulated by a lipid bilayer, serve as critical mediators of intracellular communication in both physiological and pathological states (Krylova and Feng 2023). These vesicles can influence the fate of neuroglial cells, regulate neuroinflammation, and modulate immune responses by delivering bioactive molecules such as proteins, lipids, and RNAs to target cells (Peng et al. 2020). Stem cell therapy, a prevalent therapeutic approach for SCI, confronts significant challenges including immune rejection, tumorigenic potential, and low cell survival rates (Mili and Choudhary 2024). Exosomes, presenting a safer and more controllable alternative to stem cells due to their high biocompatibility, structural stability, and lack of tumorigenic potential, have emerged as a promising substitute (Lin et al. 2025). Recent studies have underscored the therapeutic potential of exosomes in SCI. For instance, Luo et al. (2023) demonstrated that exosomes derived from adipose mesenchymal stem cells (MSCs) not only suppressed the expression of inflammatory cytokines in spinal cord tissues but also inhibited M1 microglial activation while promoting M2 polarization via the Nrf2/heme oxygenase‐1 (HO‐1) pathway. Similarly, Xue et al. (2023) observed that bone marrow MSC‐derived exosomes significantly reduced microglial M1 polarization‐mediated inflammation, demonstrating their potential to create a more reparative microenvironment. Ren et al. (2023) further highlighted that Schwann cell‐derived exosomes alleviate inflammation by driving M2 polarization, while L. Fan et al. (2022) developed an exosome‐loaded electroconductive hydrogel that effectively modulated M2 microglial polarization through the NF‐κB signaling pathway. Collectively, these findings suggest that exosome‐based therapies hold promise for harnessing microglial plasticity and promoting tissue repair following SCI.

In parallel, the intricate interplay between the gut microbiome and neuroinflammation has emerged as a compelling area of investigation. Increasing evidence supports the role of gut microbiota in influencing the CNS through the gut‐brain axis (X. Hu et al. 2023). Jing et al. (2021) demonstrated that supplementation with short‐chain fatty acids (SCFAs), metabolic byproducts of gut microbial activity, significantly improved motor recovery, enhanced neuronal survival, promoted axonal regeneration, and reduced astrocyte proliferation in a murine SCI model. These improvements were linked to the downregulation of the NF‐κB signaling pathway and the upregulation of neurotrophic factor‐3 expression. Other studies have revealed that dysbiosis of gut microbiota after intestinal barrier injury exacerbates neuroinflammation by activating microglia through the lipopolysaccharide (LPS)/TLR4/NF‐κB/NLRP3 inflammasome pathway (Cai et al. 2024). Targeting the microbiota with interventions such as SCFAs or probiotics may emerge as a novel approach to modulating detrimental microglial activation in SCI.

Another exciting frontier in SCI recovery lies in the realm of nanoparticle‐based therapies, which leverage the precision of nanoscale materials to deliver therapeutic agents directly to injury sites. Nanoparticles, by design, improve the bioavailability and targeted delivery of small‐molecule drugs, extending their residence time in affected tissues while reducing systemic side effects (Richards et al. 2024). For example, Gopalakrishnan et al. (2024) developed carbohydrate antigen‐loaded nanoparticles that not only activated microglia but also promoted their polarization toward an M2‐like reparative state. Similarly, Zhou et al. (2022) employed gold nanocluster carriers loaded with berberine to effectively inhibit M1 microglial activation while simultaneously reducing neuronal apoptosis in SCI models, thus facilitating tissue repair. These studies exemplify the versatility of nanoparticles as robust therapeutic tools to engineer the microglial response and enhance regenerative outcomes.

### 4.4 Future Directions and Actionable Recommendations

In conclusion, the field of microglial therapy for SCI continues to expand and evolve, with remarkable strides being made in regulating microglial polarization to mitigate neuroinflammation and promote recovery. Advances in exosome‐based delivery systems, gut microbiota‐targeted interventions, and nanotechnology‐based biomaterials collectively represent the forefront of SCI research. However, significant challenges remain, including the need to address scalability, optimization of therapeutic delivery, and long‐term efficacy. Bibliometrics provides data‐driven decision‐making and insights for scientific policy‐making; resource optimization allocation; and the discovery, evaluation, and prioritization of emerging treatment strategies by quantitatively analyzing large amounts of literature data, identifying research hotspots, development trends, and collaborative networks (Azizan et al. 2023; Rahayu et al. 2021; Abdullah and Azizan 2024). To address these challenges of microglia in the field of SCI and utilize established research trends, we propose the following actionable strategies:

1. Foster global collaborative networks: Bibliometric analysis revealed that China and the United States collectively contributed > 66% of publications. Notably, while the United States exhibited the highest total link strength (337), reflecting extensive international collaboration, China demonstrated disproportionately lower link strength (149) relative to its publication output. To mitigate this disparity, we recommend establishing formal international research alliances focused on microglia regulation in SCI. Such networks would facilitate data/resource sharing, personnel exchange, and coordinated multicenter clinical trials.
2. Develop integrated translational platforms: Although emerging frontiers (e.g., exosomes, gut microbiota modulation, and nanoparticles) show significant therapeutic potential, translating preclinical findings to clinical applications remains challenging. Thus, specialized translational research platforms integrating expertise in basic science, bioengineering, clinical neurology, and biostatistics are warranted to bridge this gap and accelerate practical implementation.

## 5 Limitations

This study analyzed research trends in specific fields by examining articles in the WoSCC of English‐language core journals indexed by SCIE. Although these findings offer valuable insights, several limitations should be noted.

First, this study only focused on English articles, may have overlooked articles published in other databases or languages, and lacked the inclusion of gray literature. Therefore, to avoid potential bias risks, future research should consider including databases such as PubMed or Scopus to expand the scope. Second, keyword and reference analysis may not fully reveal deeper research motivations and specific research processes. Third, the citation rate of older articles is often higher, which may make it difficult for new high‐quality literature to enter the top 10. Fourth, co‐citation analysis has limitations in reflecting the influence of new literature, mainly due to its time lag in relying on historical citation data, the continued dominance of classical literature, and the bias caused by neglecting nonacademic influence and disciplinary differences. Finally, bibliometric analysis is more effective in identifying macro‐level trends but may not be suitable for micro‐level analysis. Therefore, multimodal bibliometric content analysis methods should be adopted in the future (Zainal and Azizan 2024).

## 6 Conclusion

This bibliometric analysis comprehensively charts the significant expansion of global research on microglia in SCI over the past two decades. Our findings highlight key contributors; evolving hotspots (exosomes, microbiota, and nanoparticles); and the central, complex role of microglial‐mediated neuroinflammation. Crucially, the actionable recommendations outlined above provide a roadmap to address current challenges and accelerate the translation of this burgeoning knowledge into tangible benefits for SCI patients.

Future work must deepen our understanding of microglial mechanisms in SCI and identify novel therapeutic targets. Targeted regulation of microglial polarization to mitigate neuroinflammation holds promise as a future treatment strategy.

## Author Contributions

Wenping Lin, Ziming Cai, Qinghe Yu, and Gongpeng Xiong wrote the main manuscript text and prepared all the figures. Wenping Lin, Ziming Cai, QingheYu, Gongpeng Xiong, Jintao Wu, Hanjun Zhang, and Jian Huang were responsible for drafting the article or critically revising it for important intellectual content.

## Ethics Statement

The authors have nothing to report.

## Conflicts of Interest

The authors declare no conflicts of interest.

## Peer Review

The peer review history for this article is available at https://publons.com/publon/10.1002/brb3.70881.

## Supporting information




**Additional file 1:Supporting Table 1**: Top 15 co‐cited references related to microglia in Spinal cord injury.

## Data Availability

The data used to support the findings of this study are available from the corresponding author upon request.
