# Supplementary material for: Bibliometric Mapping of Research Trends and Hotspots of Microglia in Spinal Cord Injury (2000–2024)
Source: Brain Behav. 2025 Sep 23;15(9):e70881. doi: 10.1002/brb3.70881 (PMC12455019; doi:10.1002/brb3.70881)
Supplement: Supplementary file 1 — Additional file 1:Supporting Table 1: Top 15 co‐cited references related to microglia in Spinal cord injury. [file BRB3-15-e70881-s001.docx]

Additional file 1: Table S1: Top 15 co-cited references related to microglia in Spinal cord injury.

| **Rank** | **Author** | **Title** | **Source** | **Citation** | **Year** | **DOI** |
| --- | --- | --- | --- | --- | --- | --- |
| 1 | Kigerl, KA, et al. | Identification of Two Distinct Macrophage Subsets with Divergent Effects Causing either Neurotoxicity or Regeneration in the Injured Mouse Spinal Cord | JOURNAL OF NEUROSCIENCE | 1670 | 2009 | 10.1523/JNEUROSCI.3257-09.2009 |
| 2 | Cherry, JD, et al. | Neuroinflammation and M2 microglia: the good, the bad, and the inflamed | JOURNAL OF NEUROINFLAMMATION | 1204 | 2014 | 10.1186/1742-2094-11-98 |
| 3 | David, S, et al. | Repertoire of microglial and macrophage responses after spinal cord injury | NATURE REVIEWS NEUROSCIENCE | 1059 | 2011 | 10.1038/nrn3053 |
| 4 | DiSabato, DJ, et al. | Neuroinflammation: the devil is in the details | JOURNAL OF NEUROCHEMISTRY | 883 | 2016 | 10.1111/jnc.13607 |
| 5 | Ji, RR | Neuroinflammation and Central Sensitization in Chronic and Widespread Pain | ANESTHESIOLOGY | 745 | 2018 | 10.1097/ALN.0000000000002130 |
| 6 | Smith, JA, et al. | Role of pro-inflammatory cytokines released from microglia in neurodegenerative diseases | BRAIN RESEARCH BULLETIN | 738 | 2012 | 10.1016/j.brainresbull.2011.10.004 |
| 7 | Ransohoff, RM, et al. | Innate immunity in the central nervous system | JOURNAL OF CLINICAL INVESTIGATION | 731 | 2012 | 10.1172/JCI58644 |
| 8 | Butovsky, O, et al. | Microglia activated by IL-4 or IFN-γ differentially induce neurogenesis and oligodendrogenesis from adult stem/progenitor cells | MOLECULAR AND CELLULAR NEUROSCIENCE | 706 | 2006 | 10.1016/j.mcn.2005.10.006 |
| 9 | Rothhammer, V, et al. | Microglial control of astrocytes in response to microbial metabolites | NATURE | 662 | 2018 | 10.1038/s41586-018-0119-x |
| 10 | Davalos, D, et al. | Fibrinogen as a key regulator of inflammation in disease | SEMINARS IN IMMUNOPATHOLOGY | 660 | 2012 | 10.1007/s00281-011-0290-8 |
| 11 | Fleming, JC, et al. | The cellular inflammatory response in human spinal cords after injury | BRAIN | 645 | 2006 | 10.1093/brain/awl296 |
| 12 | Szarowski, DH, et al. | Brain responses to micro-machined silicon devices | BRAIN RESEARCH | 634 | 2003 | 10.1016/S0006-8993(03)03023-3 |
| 13 | Gaudet, AD, et al. | Wallerian degeneration: Gaining perspective on inflammatory events after peripheral nerve injury | JOURNAL OF NEUROINFLAMMATION | 613 | 2011 | 10.1186/1742-2094-8-110 |
| 14 | Shechter, R, et al. | Infiltrating Blood-Derived Macrophages Are Vital Cells Playing an Anti-inflammatory Role in Recovery from Spinal Cord Injury in Mice | PLOS MEDICINE | 598 | 2009 | 10.1371/journal.pmed.1000113 |
| 15 | Corps, KN, et al. | Inflammation and Neuroprotection in Traumatic Brain Injury | JAMA NEUROLOGY | 566 | 2015 | 10.1001/jamaneurol.2014.3558 |
